# Relative potency of a novel acaricidal compound from *Xenorhabdus*, a bacterial genus mutualistically associated with entomopathogenic nematodes

**DOI:** 10.1038/s41598-021-90726-1

**Published:** 2021-05-27

**Authors:** Gamze Incedayi, Harun Cimen, Derya Ulug, Mustapha Touray, Edna Bode, Helge B. Bode, Esra Orenlili Yaylagul, Selcuk Hazir, Ibrahim Cakmak

**Affiliations:** 1 Department of Plant Protection, Faculty of Agriculture, Aydin Adnan Menderes University, Aydin, Turkey; 2 Department of Biology, Faculty of Arts and Science, Aydin Adnan Menderes University, Aydin, Turkey; 3 Molecular Biotechnology, Department of Biosciences, Goethe Universität Frankfurt, Max-von-Laue-Str. 9, 60438 Frankfurt am Main, Germany; 4 Senckenberg Gesellschaft für Naturforschung, 60325 Frankfurt am Main, Germany; 5 Buchmann Institute for Molecular Life Sciences (BMLS), Johann Wolfgang Goethe University, Max-von-Laue-Straße 15, 60438 Frankfurt am Main, Germany; 6 Department of Natural Products in Organismic Interactions, Max-Planck-Institute for Terrestrial Microbiology, 35043 Marburg, Germany; 7 Department of Nutrition and Dietetics, Faculty of Health Sciences, Aydin Adnan Menderes University, Aydin, Turkey

**Keywords:** Zoology, Entomology

## Abstract

Our study aimed to identify the novel acaricidal compound in *Xenorhabdus szentirmaii* and *X. nematophila* using the easyPACId approach (easy Promoter Activated Compound Identification). We determined the (1) effects of cell-free supernatant (CFS) obtained from mutant strains against *T. urticae* females*,* (2) CFS of the acaricidal bioactive strain of *X. nematophila* (pCEP_kan_XNC1_1711) against different biological stages of *T. urticae,* and females of predatory mites, *Phytoseiulus persimilis* and *Neoseiulus californicus*, (3) effects of the extracted acaricidal compound on different biological stages of *T. urticae,* and (4) cytotoxicity of the active substance. The results showed that xenocoumacin produced by *X. nematophila* was the bioactive acaricidal compound, whereas the acaricidal compound in *X. szentirmaii* was not determined. The CFS of *X. nematophila* (pCEP_kan_XNC1_1711) caused 100, 100, 97.3, and 98.1% mortality on larvae, protonymph, deutonymph and adult female of *T. urticae* at 7 dpa in petri dish experiments; and significantly reduced *T. urticae* population in pot experiments. However, the same CFS caused less than 36% mortality on the predatory mites at 7dpa. The mortality rates of extracted acaricidal compound (xenocoumacin) on the larva, protonymph, deutonymph and adult female of *T. urticae* were 100, 100, 97, 96% at 7 dpa. Cytotoxicity assay showed that IC_50_ value of xenocoumacin extract was 17.71 μg/ml after 48 h. The data of this study showed that xenocoumacin could potentially be used as bio-acaricide in the control of *T. urticae;* however, its efficacy in field experiments and its phytotoxicity need to be assessed in future.

## Introduction

*Tetranychus urticae* Koch is one of the most important and widespread pest species of plant-feeding mites. It is found in tropical and temperate zones of the world especially in greenhouses. *T. urticae* uses its piercing and sucking mouthparts to aspirate contents of plant cells from a broad range of plant host species (> 1100 species). They attack several fruit and vegetables grown in greenhouses, ornamentals and field-grown crops like maize, cotton, etc.^1^. Feeding of both adult and immature stages on the lower surfaces of plant causes leaf chlorosis which eventually leads to fruit deformation, plant growth inhibition and even plant death^2^.

Generally, chemical pesticides have been used to control this mite pest, but *T. urticae* has a short life cycle and a high generative potential which has enabled it to develop resistance to various insecticides and acaricides^3^. In addition, these pesticides also kill natural enemies of *T. urticae* such as predatory mites which are biocontrol option used to suppress *T. urticae* populations^4^. The most commonly used predatory mites of *T. urticae* are *Phytoseiulus persimilis* and *Neoseiulus californicus* (Acari: Phytoseiidae)^5,6^. Because of environmental concerns and developing resistance of *T. urticae* to conventional pesticides, it is necessary to search for new acaricidal pesticides with great IPM value. Recently, several studies have investigated the effects of secondary metabolites produced by certain bacteria on insects and mites^7,8^. Recent studies have reported that some bacterial species in the genus *Xenorhabdus* produce secondary compounds with antibacterial, antifungal, nematicidal, and insecticidal properties^9–11^. These novel compounds have the potential to be developed into a new generation of pesticides including insecticides and acaricides.

*Xenorhabdus* spp. are motile, Gram-negative bacteria belonging to the family Morganellaceae^12^ and are symbiotically associated with entomopathogenic nematodes (EPNs) in the family Steinernematidae^13^. The nematode/bacterium complex has an intricate life cycle in which the bacteria are transported from one insect hemocoel to another by the steinernematid infective juveniles (IJs). The bacterial cells are sequestered in a special pouch in the intestine of the IJs. When the IJs enter their insect host through the mouth or anus and penetrate into the hemocoel or through the spiracles and penetrate directly into the hemocoel, they release the bacterial cells into the hemolymph. The highly virulent bacteria kill the insect host within 24–48 h; they also secrete enzymes, toxins and secondary metabolites with antimicrobial, insecticidal and cytotoxic activities, some of which protect the insect cadaver from saprophytic organisms^14–18^. Some of these *Xenorhabdus* compounds have potential applications in pest and disease control^9,11^. For example, numerous studies have evaluated the acaricidal activity of secondary metabolites produced by *Xenorhabdus* against several agriculturally important mite species such as *Luciaphorus perniciosus*^19,20^, *Rhizoglyphus robini*^21^, and *T. urticae*^22,23^. Moreover, Cevizci et al.^23^ reported that the egg and mobile stages of the predatory mites, *N. californicus* and *P. persimilis,* were not significantly affected by metabolites from *X. nematophila and X. szentirmaii* compared to *T. urticae.* They determined that the mode of entry of these bacterial metabolites into the mites was through the dorsal and ventral integument and that the predatory mites were less affected because of their longer legs which resulted in their less contact with the acaricide-treated surfaces.

Although the abovementioned studies have established that secondary metabolites from some *Xenorhabdus* are effective in killing mites, none was identified as to the actual compound(s) responsible for the acaricidal effects. Therefore, our study is aimed at isolating and identifying the acaricidal compound(s) using easyPACId approach (easy Promoter Activated Compound Identification)^24^. This biotechnology enables us to investigate and compare the effects of a natural products (NPs) produced by a specific gene using mutants in which the expression of the gene can be regulated^25,24^. In this approach, biological gene clusters synthesized by non-ribosomal peptide synthetases (NRPS) or polyketide synthetases (PKS) enzymes and regulated by a single promoter in *Xenorhabdus* can be activated using inducible promotors^24^.

It is important to test the effects of the new potential acaricide on the pest, *T. urticae* and its natural enemies (in this case predatory mites), to determine whether any side effects on non-target or beneficial organisms are likely to occur. Thus, the objectives of our study were to determine the (1) active acaricidal compound(s) using promoter exchange mutant strains, (2) cell-free supernatant (CFS) of the acaricidal bioactive strain of *X. nematophila* (pCEP_kan_XNC1_1711) against different biological stages of *T. urticae,* and females of predatory mites, *Phytoseiulus persimilis* and *Neoseiulus californicus*, (3) effects of the extracted acaricidal compound on different biological stages of *T. urticae,* and (4) cytotoxicity of the active substance.

## Materials and methods

### Plants

Bean plants (*Phaseolus vulgaris* cv. Barbunia supplied by Migros supermarket, Aydin, Turkey) were grown for rearing *T. urticae* as well as used for our laboratory studies. Plants were grown in pots (15 × 15 cm) containing forest soil, peat and perlite at 25 ± 2 °C temperature, 60 ± 10% relative humidity and 16 h light conditions and maintained in a separate climate room (PG34 − 3 Digitech Ltd., Ankara, Turkey) dedicated for bean plant growth.

### Rearing of mites

All mites used in the study are laboratory cultures previously identified based on morphological characteristics by Dr. Ibrahim Cakmak and used in previous studies^22,23^*. Tetranychus urticae* was obtained from strawberry plants in Aydin, Turkey. Bean plants which reached the 5–6 leaves were brought to the *T. urticae* rearing room and infested with different biological stages of the pest. The rearing of *T. urticae* was performed in another climate room with the same features as the plant growth room.

The predatory mites, *P. persimilis* and *N. californicus*, were obtained from bean plants in Hatay and strawberry plants in Aydin, respectively^6,23^. They were reared on detached bean leaves infested with all biological stages of *T. urticae* at 25 ± 1 °C temperature, 70 ± 10% relative humidity and 16 h light conditions in a third climate room. The detached bean leaves were placed on inverted pots in different size of two trays (45 × 32 × 8 cm; 78 × 56 × 18 cm). The trays were filled with water and covered with a plexiglass container to prevent the escape of the mites^26,27^. Three detached bean leaves infested with *T. urticae* were placed on each inverted pot three times a week to rear the predatory mites.

### Identification of acaricidal bioactive compounds

#### Bacterial sources

In the study carried out by Eroglu et al.^22^, *Xenorhabdus szentirmaii* and *X. nematophila* were determined as the species with the highest acaricidal activity among the CFS obtained from many tested *Xenorhabdus* and *Photorhabdus* spp. To identify the bioactive compound(s), promoter exchange mutants of *X. szentirmaii* and *X. nematophila* were generated and bioactivity tests were performed.

#### Generation of deletion and promoter exchange mutants

The easyPACId approach (easy Promoter Activated Compound Identification) was used to identify the acaricidal compound(s) in *X. szentirmaii* and *X. nematophila*. The RNA chaperon, hfq, is directly associated with the production of natural products (NPs) as it controls the expression of biosynthetic gene clusters (BGCs) using the sRNA/mRNA interactions^28^. Therefore, ∆*hfq* mutants were generated in *X. szentirmaii* and *X. nematophila* to stop the biosynthesis of NPs. Subsequently activation of desired BGCs (Table 1) in a Δ*hfq* background led to the nearly exclusive production of the corresponding NPs in *Xenorhabdus* strains following targeted BGC activation using the inducible promoter^24^. These methods can accelerate the identification of bioactive NPs by performing direct bioactivity tests without the need for purification of supernatants containing certain NPs^24,25,29^. Mutant strains of *X. szentirmaii* and *X. nematophila* with natural promoter regions replaced with inducible promoter regions were used in our study (Table 1). The generation of *X. szentirmaii* Δ*hfq* and *X. nematophila* Δ*hfq* as well as promotor exchange mutants shown in Table 1 were performed as described by Tobias et al.^28,29^ and Bode et al.^24^.

**Table 1:** Tested *Xenorhabdus szentirmaii* ∆*hfq* and *Xenorhabdus nematophila* ∆*hfq* promotor exchange mutants with the produced compound class of the selected BGC.

Strain	Analyzed BGC locus	Produced NP class
*X. szentirmaii* Δ*hfq*	*X. szentirmaii* ∆*hfq* pCEP-KM-1979	Szentirazine
	*X. szentirmaii* ∆*hfq* pCEP-KM-3460	Szentiamide
	*X. szentirmaii* ∆*hfq* pCEP-KM-3680	Xenobactine
	*X. szentirmaii* ∆*hfq* pCEP-KM-0377	PAX-short
	*X. szentirmaii* ∆*hfq* pCEP-KM-3942	Rhabduscine
	*X. szentirmaii* ∆*hfq* pCEP-KM-3397	Rhabdopeptide
	*X. szentirmaii* ∆*hfq* pCEP-KM-0346	GameXPeptide
	*X. szentirmaii* ∆*hfq* pCEP-KM-5118	Pyrrolizixenamide
	*X. szentirmaii* ∆*hfq* pCEP-KM-fclC	Fabclavine
	*X. szentirmaii*_pBADxpzA	Phenazine
	*X. szentirmaii*_pBADxpzV	Iodinine
	*X. szentirmaii*_pBADxpzI	Phenaszentine
*X. nematophila* Δ*hfq*	*X. nematophila* Δ*hfq* pCEP-KM-XNC1-2783	PAX-peptide
	*X. nematophila* Δ*hfq* pCEP-KM-XNC1-2040	Xenoamicine
	*X. nematophila* Δ*hfq* pCEP-KM-XNC1-1711	Xenocoumacin
	*X. nematophila* Δ*hfq* pCEP-KM- XNC1-2300	Xenortide
	*X. nematophila* Δ*hfq* pCEP-KM- XNC1-2022	Xenotetrapeptide
	*X. nematophila* Δ*hfq* pCEP-KM- XNC1-2228	Rhabdopeptide
	*X. nematophila* Δ*hfq* pCEP-KM- XNC1-2713	Xenematide
	*X. nematophila* Δ*hfq* ΔisnAB pBAD-XNC1-2300	Xenortide
	*X. nematophila* ΔPPTase pBAD-XNC1-isnA	Rhabduscin

#### Preparation of bacterial supernatants of mutant strains

The 21-promoter exchange mutant strains (12 *X. szentirmaii* and 9 *X. nematophila*) listed in Table 1 were cultivated on LB agar, supplemented with a 50 μg/mL final concentration of kanamycin, and incubated for 48 h at 30°C^30^. A single colony was transferred into 10 ml LB medium, supplemented with a 50 μg/ml final concentration of kanamycin to obtain an overnight culture at 200 rpm and 30 °C. The optical densities of the overnight cultures (10 ml LB) were measured at 600 nm. The final OD of the cultures was adjusted to 0.1 after inoculation 100 ml Nutrient Broth (NB)^30^. For each strain, two flasks were prepared, and the cultures were incubated at 30 °C for 1 h. One of the flasks was induced with 0.2% L-arabinose (Carl Roth), and the other flask was not treated with L-arabinose (non-induced). All induced and non-induced cultures were incubated for 72 h at 200 rpm and 30 °C. The CFS was harvested by centrifugation at 10,000 rpm for 10 min, and the supernatant was filtered through a 0.22 μm millipore filter (Thermo scientific)^10,31^.

#### Determination of acaricidal compound/s using mutant strains

The effects of induced and non-induced CFS of mutant strains were tested on *T. urticae* adult females in Petri dishes. Experiments were carried out in a climate room (PG34 − 3 Digitech Ltd., Ankara, Turkey) at 25 ± 1 °C temperature, 70 ± 5% relative humidity and 16 h light conditions. Moistened cotton wool was placed on Petri dishes (15 cm in diameter) first, and then the bean leaf was placed with its bottom face up. The adult females of *T. urticae* were separately transferred with a fine brush in each Petri dish as 20 individuals. The CFS of mutant strains were sprayed on the leaves with a hand sprayer (2.5 ml/Petri dish). Sterile NB medium in which bacteria were grown was used as the control group. Mortality rates of mites were determined in 2, 5 and 7 days after the application (dpa). The experiments were carried out in 20 repetitions and repeated 4 times at different times.

### The effect of the supernatant of induced mutant strain responsible from acaricidal activity against different biological stages of *Tetranychus urticae*

The acaricidal compound that causes high mortality on mites was determined and the gene region responsible for the production of the relevant bioactive compound in *X. nematophila* (pCEP_kan_XNC1_1711) was induced by L-arabinose, thus enabling the bacterium to produce an acaricidal bioactive compound only as a secondary metabolite. The effects of this CFS against the different biological stages of *T. urticae* were investigated in Petri dishes and pots.

### Petri dish experiments

The effects of CFS of *X. nematophila* pCEP_kan_XNC1_1711 against different biological stages (egg, larva, protonymph, deutonymph, adult) of *T. urticae* were detected as previously described. Moistened cotton wool was placed on Petri dishes (15 cm in diameter) first, then the bean leaf was placed with its bottom face up. The egg, larva, protonymph, deutonymph and adult female of *T. urticae* were separately transferred with a fine brush in each Petri dish as 20 individuals. In order to obtain different biological stages of *T. urticae* at the same age to be used in the experiments, 25 gravid females of *T. urticae* were transferred on leaf discs. After 24 h, females were removed from the environment and the eggs remained. In this way, different biological stages (egg, larva, protonymph, deutonymph and adult female) of *T. urticae* were obtained at the same age. The CFS of mutant strain was sprayed on the leaves with a hand sprayer (2.5 ml/Petri dish). Sterile NB was used as the control group. Mortality rates of mites were determined in 2, 5 and 7 dpa. The experiments were carried out in 20 repetitions and repeated 4 times at different dates.

### Pot experiments

As in Petri dish experiments, bean plants were used in pot experiments. Bean plants were grown in pots (7 × 5 cm) and used at the same age in the experiment. One leaf of the plants with two cotyledon leaves was cut, and only one leaf was left per each pot. A total of 60 individuals, including 10 individuals of each stage, egg, larva, protonymph, deutonymph, adult female and adult male, obtained from *T. urticae* culture were transferred to these plants with a fine brush. The CFS of *X. nematophila* pCEP_kan_XNC1_1711 mutant strain was sprayed on both the bottom and top surfaces of the leaves with a hand sprayer (5 ml/pot). The plants in the control group were sprayed with the same amount of sterile NB. The number of live and dead individuals was recorded 7 dpa. The experiments were carried out in 20 repetitions and repeated 4 times at different dates.

### The toxicity of the supernatant of mutant strain on predatory mites

The potential toxic effect of the CFS of *X. nematophila* pCEP_kan_XNC1_1711 on the predatory mites, *P. persimilis* and *N. californicus*, was investigated in Petri dishes at 25 ± 1 °C, 70 ± 5% R.H. and 16 h L:D photoperiod in a climate room. Moistened cotton wool (10 cm diameter) was placed on the Petri dishes (15 cm in diameter), and the gap between the Petri dish and cotton was filled with tap water to prevent the escape of the predatory mites. Adult females of *P. persimilis* and *N. californicus* (20 individuals/Petri dish) obtained from the culture were separately transferred with a fine brush on the leaves in the Petri dishes. Bean leaves infested with different biological stages of *T. urticae* (~ 300 individuals) were brushed onto the leaves at two-day intervals to feed the predatory mites. The CFS of mutant strain was sprayed with a hand sprayer on the leaves, and sterile NB was used as control. The mortality rate of the predatory mites in each Petri dishes was recorded at 2, 5 and 7 dpa. The study was carried out in 20 repetitions and repeated 4 times at different times.

### The effects of the acaricidal extract on different biological stages of *Tetranychus urticae*

Extraction of the acaricidal bioactive compound was performed as follows:

Induced *X. nematophila* pCEP_kan_XNC1_1711 mutant strain was cultured in LB (6L) with 2% XAD resin at 30 °C for 3 days. The resin was extracted exhaustively with methanol (3 × 2 L) at room temperature. The methanol extract was concentrated under reduced pressure to give extracted compound^24^. The obtained extracted compound was first dissolved in DMSO and prepared as a stock solution with distilled water at a concentration of 208 μg/ml. Different dilutions (100%, 50%, 25%, 12.5%, 6.25% and 3.125%) of this prepared stock solution to determine LC_50_ and LC_90_ value of extracted compound were applied to *T. urticae* females in Petri dishes. Then, the activity of the LC_90_ value of the acaricidal extracted compound on different biological stages (egg, larva, protonymph, deutonymph and adult female) of *T. urticae* was determined in Petri dishes. For these studies, moistened cotton wool was placed on Petri dishes (15 cm in diameter) first, then the bean leaf was placed with its bottom face up. 20 adult females of *T. urticae* were transferred to each petri dish with a fine brush. Different dilutions and the LC_90_ value of the extracted compound were sprayed on the leaves with a hand sprayer (2.5 ml / petri dish). Sterile distilled water with DMSO was used as the control group. Mortality rates of mites were determined in 2, 5 and 7 dpa. The experiments were carried out in 10 repetitions and repeated 2 times.

### Cytotoxicity of extracted bioactive acaricidal compound

Cytotoxicity assay was conducted using MRC-5 normal human fetal lung fibroblast cell-line. MRC-5 cells were obtained from the cell culture bank of the Turkish Ministry of Agriculture and Forestry (MRC-5 An_1_, HÜKÜK no: 96101701). The cells were maintained in Eagle's Minimum Essential Medium (EMEM) supplemented with 10% fetal bovine serum (Sigma-Aldrich) and 5% penicillin–streptomycin solution. The cells were cultured in tissue culture flask and incubated at 37 °C, 5% carbon dioxide and 96% humidity. The culture medium was replenished in 2 day-intervals. The cytotoxic effects of the extracted acaricidal compound of *X. nematophila* pCEP_kan_XNC1_1711 were measured in MRC-5 cell line using the MTT method. MRC-5 cells were treated with various concentrations of extracted acaricidal compound for 48 h at 37 °C. The compound was first dissolved in Dimethyl sulfoxide (DMSO) and prepared as a stock solution with distilled water at a concentration of 208 µg/ml. Six different concentrations ranging from 1.04 to 72.8 µg/ml and 2.08 to 104 µg/ml were prepared in EMEM (Sigma-Aldrich) respectively. MRC-5 (1 × 10^4^cells/well) were seeded in each well of 48-well microplates and incubated at 37 °C and 5% CO_2_ for 24 h. Then, extracted acaricidal compound were applied and the cells were incubated for 48 h. There were two control groups: one with culture medium and MRC-5 cells and the other had DMSO solvent. After 48 h, the MTT [3-(4,5-Dimethylthiazol-2-yl)-2,5-Diphenyltetrazolium Bromide] solution (5000 µg/ml) was added to each well, and the cells were cultured for another 4 h at 37 °C in a 5% CO_2_ incubator^32^. A hundred microliters of DMSO was added to the cells to dissolve the formazan crystals that formed. After 15 min of mixing at room temperature, the level of colored formazan was determined by measuring optical density (OD) with Multiskan™ GO Microplate reader (Thermo Scientific™, Finland) at 570 nm (OD_570-630 nm_)^33^. The half-maximal inhibitory concentrations (IC_50_) values were measured after 48 h. The assays were performed in three independent experiments. The percentage viability was calculated as^34^: % Viability = (OD of treated cells/OD of control cells) × 100.

### Statistical analyses

**Figure 1:**
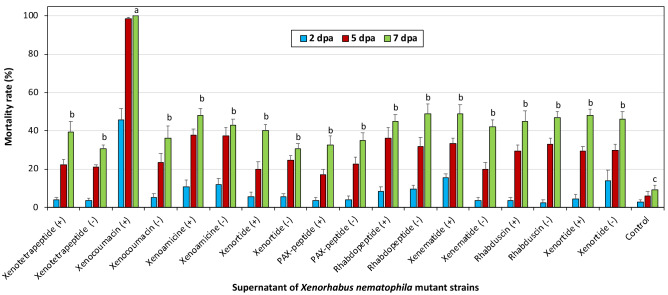
The mortality of induced or non-induced mutant strains of *X. nematophila X. nematophila* on *Tetranychus urticae* females.

The data shown in Figs. 1, 3 and 6 were analyzed with the General Linear Model and the differences among the averages were grouped according to the Tukey’s Honestly Significant Difference (Tukey HSD) test at the level of P = 0.05. The data obtained in Figs. 3 and 6 were calculated by applying the Abbott formula^35^. The data in Figs. 4 and 5 were compared with Student’s *t*-test. Arcsine transformation was performed on mite mortality before statistical analyses^36^. The LC_50_ and LC_90_ values were determined in the POLO computer package program^37^.

### Ethical statements

The authors declare that the use of bean plants in the present study complies with international, national and/or institutional guidelines. Bean plants, *Phaseolus vulgaris* cv. Barbunia were supplied by Migros supermarket, Aydin, Turkey.

## Results

### Determination of acaricidal compound/s using mutant strains

**Figure 2:**
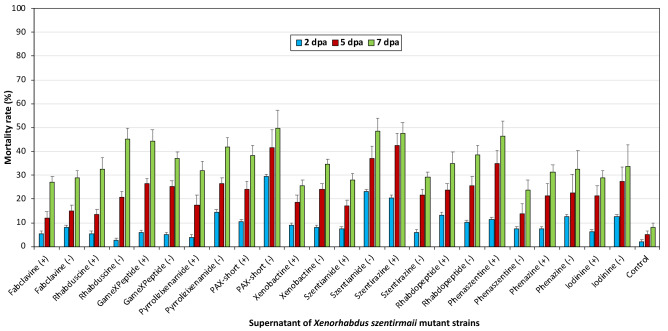
The mortality of induced or non-induced mutant strains of *X. szentirmaii* on *Tetranychus urticae* females.

The experiments conducted with mutant strains showed that xenocoumacin induced strain of *X. nematophila* (pCEP_kan_XNC1_1711) exhibited the highest acaricidal effect on *T. urticae* (Fig. 1). When the gene region responsible for the production of xenocoumacin was induced, the mortality rate of mites at 7 dpa was 100%, while the mortality rate of the non-induced xenocoumacin gene was less than 40%. None of the other induced or non-induced mutant strains of *X. nematophila* caused more than 50% mortality at 7 dpa. There was a statistically significant difference between xenocoumacin and all of the other tested compounds and the control group (F = 16.695, df = 18, P < 0.001) (Fig. 1). On the other hand, induced or non-induced 12 mutant strains of *X. szentirmaii* displayed acaricidal activity less than 50% (Fig. 2).

### The effect of the supernatant of induced mutant strain responsible from acaricidal activity against different biological stages of *Tetranychus urticae*

#### Petri dish experiments

**Figure 3:**
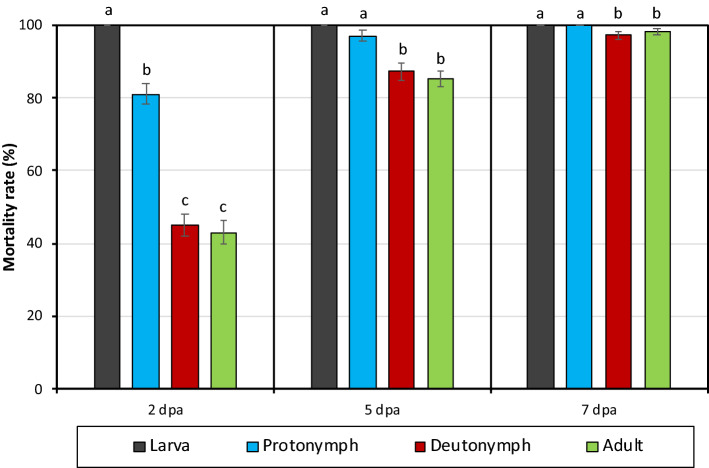
The effects of cell free supernatant produced by induced *Xenorhabdus nematophila* (pCEP_kan_XNC1_1711) mutant strain on different biological stages of *Tetranychus urticae* in Petri dish.

The study showed that the CFS of *X. nematophila* (pCEP_kan_XNC1_1711) mutant strain had no effect on *T. urticae* eggs (ovicidal rate was 0%). The mortality rates on larva, protonymph, deutonymph and adult female of *T. urticae* were 100, 81, 44.9, and 43.1% at 2 dpa (Fig. 3). There was a statistical difference in mortality rates between different biological stages of *T. urticae* at 2 dpa, and the highest mortality rate was found in larvae (F = 187,580; P < 0.001). The highest mortality was detected in larvae and protonymphs at 5 and 7 dpa, and the lowest was in deutonymphs and adults (5 dpa F = 24.417, P < 0.001; 7 dpa F = 4.694, P < 0.05; Fig. 3). The mortality rate in all biological stages of *T. urticae* was over 85% at 5 dpa and over 97% at 7 dpa (Fig. 3).

#### Pot experiments

**Figure 4:**
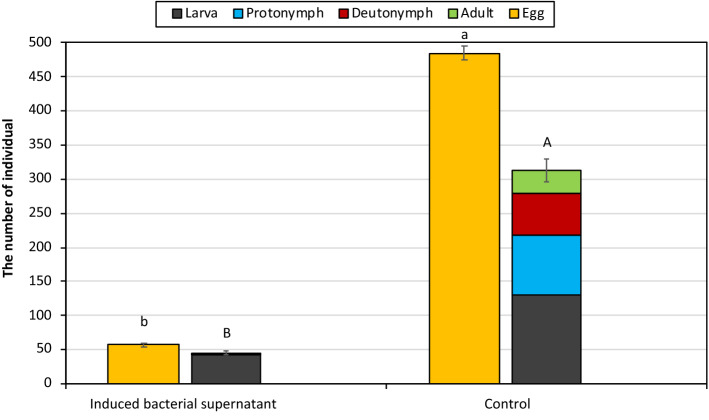
The effects of cell free supernatant produced by induced *Xenorhabdus nematophila* (pCEP_kan_XNC1_1711) mutant strain on different biological stages of *Tetranychus urticae* in pot experiments.

**Figure 5:**
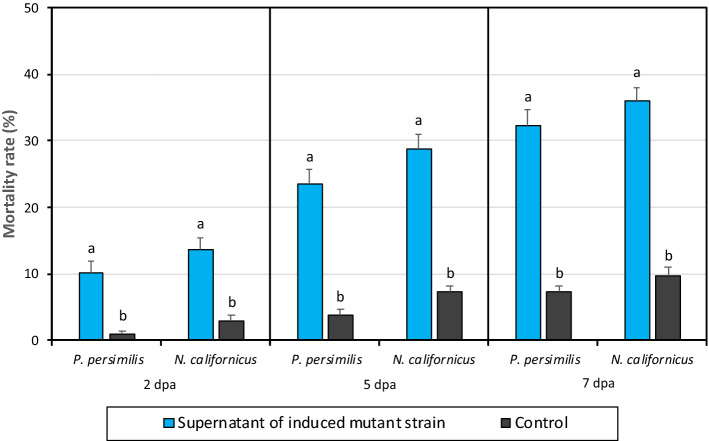
The toxicity of cell free supernatant produced by induced *Xenorhabdus nematophila* (pCEP_kan_XNC1_1711) mutant strain on adult female of *Phytoseiulus persimilis* and *Neoseiulus californicus*.

The number of live individuals at 7 days after the application of the CFS to the different biological stages of *T. urticae* is shown in Fig. 5. At 7 dpa, 57 and 485 eggs and 45 (42.4 larvae, 0.1 protonymph, 0.6 deutonymph, 1.9 adult) and 313 (130.6 larvae, 87 protonymph, 60.6 deutonymphs, 34.9 adults) mobile stages were obtained in CFS treated group and control, respectively. The number of both eggs and mobile stages was significantly different between the CFS and the control (eggs t = 42.988, P < 0.01; mobile stages t = 41,307, P < 0.01) (Fig. 4).

### The toxicity of the supernatant of mutant strains on predatory mites

The mortality rates at 2, 5 and 7 dpa of the CFS of *X. nematophila* (pCEP_kan_XNC1_1711) mutant strain to the adult stages of the predatory mites *P. persimilis* and *N. californicus* are given in Fig. 5. The mortality rate of adult females of *P. persimilis* at 2, 5 and 7 dpa was 10.3, 23.5, 32.3% in the CFS treated group and 1, 3.8, 7.3% in the control, respectively. There was a statistically significant difference between the CFS and the control (2 dpa t = 5.54, P < 0.01; 5 dpa t = 8.454, P < 0.001; 7 dpa t = 9.499, P < 0.001) (Fig. 5). The mortality rate of adult females of *N. californicus* at 2, 5 and 7 dpa was 13.8, 28.8, 36.0% in the CFS treated group and 3, 7.3, 9.8% in the control, respectively. Statistically significant difference was observed between the CFS and the control groups (2 dpa t = 5.669, P < 0.05; 5 dpa t = 9.284, P < 0.01; 7 dpa t = 11.132, P < 0.01) (Fig. 5). However, no significant difference occurred between *P. persimilis* and *N. californicus* in terms of sensitivity to the CFS (2 dpa t = 1.520, P > 0.05; 5 dpa t = 1.752, P > 0.05; 7 dpa t = 1.193, P > 0.05).

### The effects of the extracted acaricidal compound on different biological stages of *Tetranychus urticae*

**Figure 6:**
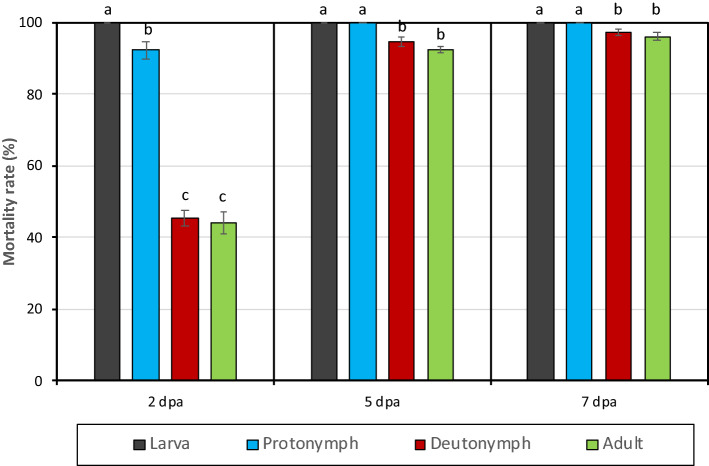
The effects of the extracted acaricidal compound (xenocoumacin) on different biological stages of *Tetranychus urticae* in Petri dish.

The data revealed that xenocoumacin is an extremely effective acaricidal compound. Even a 25% concentration of xenocoumacin caused death by 93.8% of *T. urticae* adult females at 7 dpa. Because of DMSO, control mortalities were ranged between 1.3 (2 dpa) and 15.0% (7 dpa) (Table 2). The LC_50_ values of xenocoumacin for 2, 5 and 7 dpa were calculated as 60, 26, 21 µg/ml and the LC_90_ values as 301, 71, 55 µg/ml, respectively. When the LC_90_ value (71 µg/ml) in 5 days was applied to the different biological stages of *T. urticae*, the mortality rates of xenocoumacin on the larva, protonymph, deutonymph and adult female of *T. urticae* were 100, 92, 45, 44% at 2-dpa, 100, 100, 94, 92% at 5 dpa and 100, 100, 97, 96% at 7 dpa (Fig. 6). There was a statistically significant difference in the mortality rates of different biological stages of *T. urticae* at 2, 5 and 7 dpa (2 dpa F = 169.005; P < 0.001; 5 dpa F = 32.665 P < 0.001; F = 9.717; P < 0.001; Fig. 6). On the other hand, xenocoumacin had no effect on the egg stages of *T. urticae* (ovicidal rate 0%).

**Table 2:** Effect of different dilutions of extracted acaricidal compound (xenocoumacin) on *Tetranychus urticae* adult females (mean ± S.E.).

Tested compound	Concentrations (μg/ml)	Mortality rate after application (%)		
		2 dpa	5 dpa	7 dpa
Xenocoumacin	208	95.0 ± 2.9	100.0 ± 0.0	100.0 ± 0.0
	104	46.3 ± 5.5	93.8 ± 3.8	96.3 ± 2.4
	52	46.3 ± 6.9	85.0 ± 3.5	93.8 ± 3.8
	26	30.0 ± 5.4	51.3 ± 9.4	62.5 ± 6.0
	13	13.8 ± 7.7	22.5 ± 7.8	28.8 ± 7.5
	6.5	7.5 ± 3.2	20.0 ± 4.6	30.0 ± 2.0
Control (distilled water with DMSO)		1.3 ± 1.3	13.8 ± 2.4	15.0 ± 4.6

### Cytotoxity of extracted acaricidal bioactive compound

Cytotoxicity of extracted acaricidal bioactive compound (xenocoumacin) increased with increasing concentration. The inhibitory concentration (IC_50_) value after 48-h incubation was 17.71 μg/ml.

## Discussion

When entomopathogenic nematodes infect an insect host, their symbiotic bacteria produce a wide variety of biologically active compounds with a broad-spectrum activity to protect infected cadaver from opportunistic organisms and scavengers such as ants, crickets, cockroaches, mites etc.^38,39^. It has been reported that mites like *Hypoaspis* sp., *Pergamasus nr. crassipes* (Mesostigmata), *Eugamasus* sp. (Mesostigmata), *Cosmolaelaps vacua, Ololaelaps veneta*, *Gamasellodes vermivorax, Antennoseius* sp., *Amblyseius setulus, Ascanesoica, Alycus roseus*, *Pilogalumna cozadensis* (Oribatida), *Alicorhagia fragilis* (Endeostigmata), *Tyrophagus putrescentiae* (Astigmata) prey on EPN IJs^40^. Moreover, species like *Sancassania polyphyllae* (Astigmata) have been observed to feed on EPN-infected insect cadavers and on the developing EPN IJs herein^41–46^. To protect infected cadaver and developing nematodes from mites, nematode-bacteria complex has to produce bioactive acaricidal compound/s. Accordingly, numerous studies have shown that some species of *Xenorhabdus* bacteria have acaricidal activity^19–23^. However, none of these studies identified the bioactive acaricidal compound. Therefore, the aim of this study was to establish the acaricidal activities present in *X. nematophila* supernatant using the easyPACId biotechnological approach. This biotechnological approach allowed us to determine the bioactive compound by activating mutants with inducible promotors of encoding gene clusters and eliminating the background effect of genes of other compounds^24,25^. The experiments conducted with promoter exchange mutant strains showed that xenocoumacin induced strain of *X. nematophila* (pCEP_kan_XNC1_1711) exhibited the highest acaricidal effect on *T. urticae*.

Xenocoumacins are benzopyran-1-one (isocoumarin) derivatives first identified by Mclnernery et al.^47^ in *X. nematophila* in two forms. Reimer et al.^15^ later discovered 4 additional derivatives of these natural products system from several *Xenorhabdus* strains and reported that they are synthesized by a hybrid polyketide synthase (PKS)‐nonribosomal polypeptide synthetase (NRPS). Both forms have many biological activities such as antifungal, antibacterial, anticancer and anti-ulcer however, xenocoumacin 1 is more biologically active^11,48,49^.

On the other hand, induced or non-induced 12 mutant strains of *X. szentirmaii* displayed acaricidal activity less than 50%. A large-scale genome and metabolome analysis of 25 *Xenorhabdus* strains by Tobias et al.^29^ revealed that *X. szentirmaii* DSM 16,338 and US strains do not produce xenocoumacin. So, the acaricidal compound must be a different compound than xenocoumacin. Our collection of mutant strains from *X. szentirmaii* in our study was limited. Further studies should be conducted with different promotor exchange mutants of *X. szentirmaii.*

We assessed the acaricidal effects of CFS of *X. nematophila* (pCEP_kan_XNC1_1711) against all biological stages of an important argonomic pest, *T. urticae*. First, we showed that the mobile stages of *T. urticae* were affected at different levels by the CFS of *X. nematophila* (pCEP_kan_XNC1_1711) mutant strain and xenocoumacin extract. Larval stages were more susceptible compared to adult female in Petri dish experiments, though the mortality rate in all biological stages of *T. urticae* was over 97% at 7 dpa. Similarly, Eroglu et al.^22^ found that female adults were relatively more tolerant to the supernatants of *X. nematophila* wildtype than larval and nymph stages as the supernatant exhibited 90% mortality on adult females and 98% mortality on the larvae of *T. urticae* at 7 dpa. The LC_50_ values of xenocoumacin extract against *T. urticae* adult females in our study for 2, 5 and 7 dpa were 60, 26, 21 µg/ml, respectively. Comparatively, Furuya et al.^50^ reported that a novel acaricidal compound, pyflubumide, had a LC_50_ value of 1.2 mg a.i./L against adult twospotted spider mites. The LC_50_ for cyflumetofen against *T*. *urticae* female adults as reported in Hayashi et al.^51^ was 1.1 mg/L.

Besides petri dish experiments, the results of our pot experiment showed that CFS of *X. nematophila* (pCEP_kan_XNC1_1711) mutant strains significantly reduced the *T. urticae* population. Likewise, Eroglu et al.^22^ showed that the supernatants from wildtypes of *X. szentirmaii* and *X. nematophila*, singularly and in combination, significantly reduced the *T. urticae* population in pot experiment.

We also tested the effects of CFS of *X. nematophila* (pCEP_kan_XNC1_1711) mutant strains against eggs of *T. urticae.* We found that xenocoumacin had no effect on *T. urticae* eggs (ovicidal rate was 0%). Generally, mite eggs have been observed to be resistant to acaricide^52^, supernatants of *Xenorhabdus* and *Photorhabdus* bacteria^22,23^ or infection from entomopathogenic fungi^52^.

*Tetranychus urticae* is the most resistant species among arthropod pests in the world as it has gained resistance to 96 currently available active ingredients^53^. Hence, predatory mites like *P. persimilis* and *N. californicus* are widely used as alternatives to control *T. urticae* populations. An ideal acaricidal compound should kill *T. urticae* and have minimum side effects on these predatory mites. Our study also evaluated the toxicity of CFS of *X. nematophila* (pCEP_kan_XNC1_1711) against the adult females of *P. persimilis* and *N. californicus.* Although, the CFS of *X. nematophila* (pCEP_kan_XNC1_1711) or xenocoumacin XAD extract caused over 90% mortality on the adult female of *T. urticae,* less than 40% mortality of both predatory mites were affected at 7dpa. Morphological differences between predatory mites and *T. urticae* have a key role in the different susceptibility of the mites to bacterial supernatants. For instance, *T. urticae* feeds on treated leaves and have shorter legs compared to predatory mites, their body parts are more in direct contact with applied compounds^23^. Besides this, predatory mites have a thicker cuticle than of *T. urticae*^54^.

Cytotoxicity assays revealed that xenocoumacin compound is not toxic on human cells when it is used at concentrations < 17.71 μg/ml. Bode et al.^24^ tested the effect of aqueous extract of xenocoumacin obtained from *X. nematophila* on the human microvascular endothelial cell line. Except for toxicity on cell proliferation, xenocoumacin extract displayed very low effect on the cell metabolic activity. Cytotoxicity of xenocoumacin was moderate and leucocyte adhesion to endothelial cell was low. We found that the LC_50_ values of xenocoumacin extract against *T. urticae* adult females in our study ranged between 21–60 µg/ml during the 7 days of assessment. However, this is higher than cytotoxicity against human cells. Future studies should assess the persistence of this compound on plant tissues.

In conclusion, the data of this study showed that xenocoumacins could potentially be used as bio-acaricides in the control of *T. urticae* at concentrations less than 17 μg/ml*,* however, the efficacy of xenocoumacin in the field experiment and its phytotoxicity need to be assessed in future.
